# Dual case insights shaping a new protocol for post-menopausal hirsutism

**DOI:** 10.1210/jcemcr/luag087

**Published:** 2026-05-06

**Authors:** Einas Mohamed, Jemima Sellicks, Amy Kitchener, Amy E Morrison, Miles J Levy, Karim Meeran

**Affiliations:** Division of Diabetes, Endocrinology and Metabolism, Section of Endocrinology and Investigative Medicine, Imperial College London, London W12 0NN, UK; Department of Endocrinology, University Hospitals of Leicester NHS Trust, Leicester LE1 5WW, UK; Department of Histopathology, University Hospitals of Leicester NHS Trust, Leicester LE1 5WW, UK; Department of Endocrinology, University Hospitals of Leicester NHS Trust, Leicester LE1 5WW, UK; Department of Endocrinology, University Hospitals of Leicester NHS Trust, Leicester LE1 5WW, UK; Division of Diabetes, Endocrinology and Metabolism, Section of Endocrinology and Investigative Medicine, Imperial College London, London W12 0NN, UK

**Keywords:** hyperthecosis, hyperandrogenism, androgen-secreting ovarian tumors, hirsutism, GnRH antagonist

## Abstract

Hyperandrogenism in women typically presents with hirsutism and other virilizing features. When it presents with accelerated clinical features and/or high androgen levels (total testosterone > 5 nmol/L) (>145 ng/dL) (reference range, <2.4 nmol/L [SI: <70 ng/dL]), particularly in the post-menopausal state, an underlying ovarian or adrenal neoplasm should be considered. With ovarian pathology, androgen-secreting ovarian tumors and ovarian hyperthecosis are the key differential diagnoses. The recommended treatment for androgen-secreting ovarian tumors (ASOTs) is surgical removal. In contrast, ovarian hyperthecosis may be managed conservatively, provided an androgen-secreting malignant tumor has been excluded. Clarifying this diagnostic uncertainty is a challenge, and imaging and biochemical tests are not always diagnostic. There is additionally a need for better medical treatment in patients with benign ASOTs. We report two cases of female hyperandrogenism with a radiologically identified ovarian lesion, managed with a gonadotropin-releasing hormone (GnRH) antagonist, namely, degarelix. Degarelix was helpful for both diagnosis and treatment of symptoms. We highlight its potential role in diagnosis and medical management, particularly as an option for patients who are unwilling or unable to undergo immediate surgery. We also hypothesize that a fall in total testosterone following GnRH antagonist injection may indicate that an ovarian tumor is benign.

## Introduction

By far the commonest cause of androgen excess in women is polycystic ovarian syndrome (PCOS). However, more pronounced clinical features associated with total testosterone levels >5 nmol/L (SI: >145 ng/dL) (reference range, <2.4 nmol/L [SI: <70 ng/dL]), and elevation in other androgens raise suspicion of a potentially underlying adrenal or ovarian tumor [1]. Approximately one percent of ovarian tumors are androgen-secreting ovarian tumors (ASOTs), arising from sex cord and stromal cells of the ovary. Sertoli-Leydig cell tumors are non-epithelial germ cell tumors, accounting for less than 0.5% of all ovarian neoplasms. Around 50% of Sertoli-Leydig cell tumors are hormone-secreting, producing predominantly androgens or, less commonly, estrogens [2]. Ovarian hyperthecosis describes luteinized theca cells, scattered throughout the ovarian stroma. These nests of cells secrete androgens and tend to give higher total testosterone levels and more severe androgenic symptoms than those seen in PCOS [3, 4].

Baseline androgen levels can help distinguish adrenals from ovarian sources. Elevated dehydroepiandrosterone sulfate (DHEAS) and 17-hydroxyprogesterone (17-OHP), with or without dexamethasone suppression, may suggest an adrenal origin. Isolated total testosterone elevation favors an ovarian source, and if there is an ovarian mass, a diagnosis could be made on clinical and radiological grounds. There is increasing use of urine and saliva steroid metabolites to further stratify whether androgens are likely to have an adrenal or ovarian source. Where there is uncertainty, suppression of total testosterone by more than 50% of baseline with a gonadotropin-releasing hormone (GnRH) analog implies an ovarian source [5]. There is a need for better confirmatory non-invasive tests in the context of post-menopausal virilization. When a unilateral ovarian mass is confirmed on imaging, surgical excision remains the current standard treatment [6]. Not all patients are able or willing to undergo surgical management, highlighting a need for improved medical therapies.

GnRH antagonists such as degarelix rapidly suppress gonadotropins without the initial flare seen with GnRH analogs. This suppression of luteinizing hormone (LH) rapidly switches off gonadotropin-stimulated androgen production. Their primary use has been in prostate cancer, but we propose their use in managing women with ovarian hyperandrogenism. Current literature describes the use of GnRH analogs, rather than antagonists, in the diagnosis and treatment of women with hyperandrogenism. We present two cases of women with androgen-secreting ovarian tumors presenting with significantly elevated total testosterone levels, where the GnRH antagonist, degarelix, was helpful in the diagnosis and treatment. We suggest a possible new protocol for the investigation of women with suspected ovarian virilization [7].

## Case presentations

### Case 1

A 77-year-old woman presented with a several-month history of progressive hirsutism and voice deepening. Physical examination revealed significant facial and body hirsutism, deepened voice, and no overt signs of virilization beyond these features, specifically no clitoromegaly. She had a history of colon cancer, hypertension, and type 2 diabetes.

### Case 2

A 45-year-old woman presented with secondary amenorrhea, accelerated hirsutism, acne, and irritability. Physical examination revealed moderate-to-severe hirsutism. No clitoromegaly or other virilizing signs were present. She has a past medical history of B-cell lymphoma involving a thymus gland resection.

## Diagnostic assessment

### Case 1

**Figure 1:**
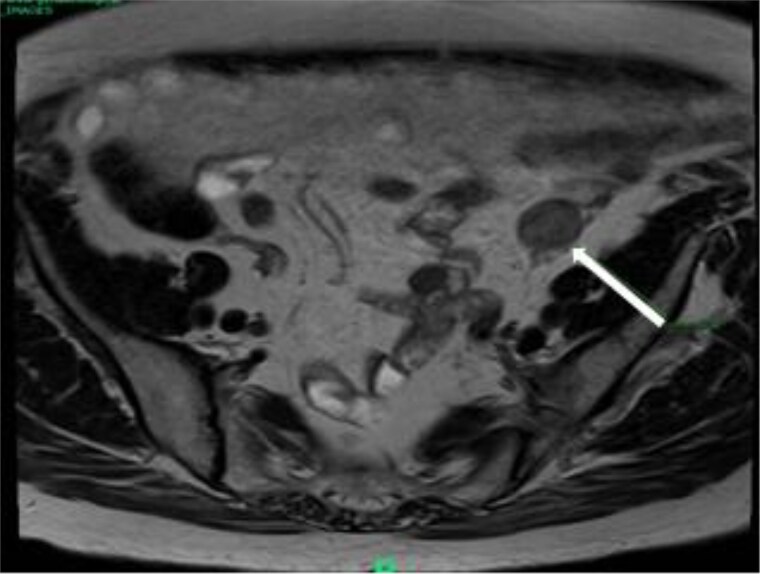
T1-weighted magnetic resonance imaging of the pelvis demonstrating a left adnexal mass in Case 1. The arrow points to the mass.

Laboratory investigations revealed significantly elevated total testosterone levels 14 nmol/L (SI: 404 ng/dL), with non-suppressed LH and follicle-stimulating hormone (FSH), as well as polycythemia. DHEAS was within normal range. Pelvic magnetic resonant imaging (MRI) identified a solid enhancing lesion in the left adnexa, suggestive of an ovarian mass (Fig. 1). The likely diagnosis was felt to be an ASOT or ovarian hyperthecosis.

### Case 2

**Figure 2:**
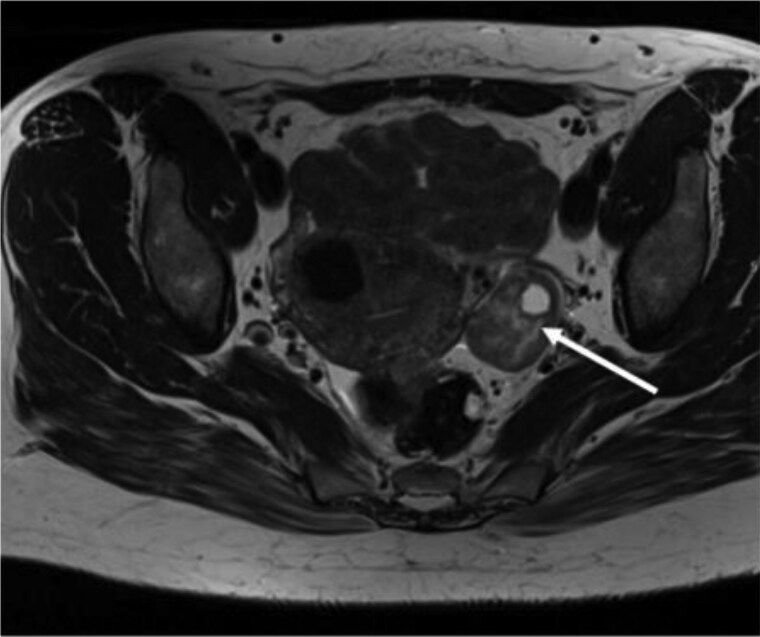
T1-weighted magnetic resonance imaging of the pelvis demonstrating a left adnexal mass in Case 2. The arrow points to the mass.

Initial investigations revealed significantly elevated total testosterone 6.6 nmol/L (SI: 190 ng/dL), with normal adrenal androgen precursors and non-suppressed LH and FSH. Pelvic ultrasound, followed by MRI, revealed a 3 cm left adnexal mass (Fig. 2). This was initially reported as a broad ligament fibroid, but in view of the clinical presentation, it was felt more likely to be a Leydig or thecal cell tumor. 18F-fluorodeoxyglucose positron emission tomography-computed tomography (FDG-PET-CT) demonstrated no increased uptake in the adnexal mass, suggesting benign pathology.

## Treatment

### Case 1

The multidisciplinary gynecology-oncology team recommended laparoscopic bilateral salpingo-oophorectomy with omental and peritoneal biopsies. The patient declined surgery. A single dose of GnRH antagonist (degarelix 80 mg) was administered to assess testosterone suppression and therapeutic response. There was marked suppression in total testosterone levels; within twenty-four hours, it decreased from 14 nmol/L (SI: 404 ng/dL) to 1.5 nmol/L (SI: 43 ng/dL) and fell below 1.0 nmol/L (SI: < 29 ng/dL) within forty-eight hours (Table 1). This response was suggestive of a benign disease. The patient subsequently received monthly degarelix injections from October 2023 to December 2024 (15 doses), followed by 3-monthly dosing during 2025 (five doses to date).

**Table 1:** Hormone laboratory values pre-treatment, during treatment, and up to 72 hours post treatment with GnRH Antagonist (Degarelix).

Time after GnRH Antagonist administration (hours)		Total testosterone (nmol/L [SI]; ng/dL [US])	LH (IU/L [SI]; mIU/mL [US])
		*0.5-2.6 nmol/L (≈ 14-75 ng/dL)*	*15.9-54.0 IU/L (≡ 15.9-54.0 mIU/mL)*
**Case 1**	Baseline	**14 nmol/L (∼404 ng/dL)**	24 IU/L; mIU/mL
2 hours		—	—
4 hours		**7.5 nmol/L (∼216 ng/dL)**	10.2 IU/L; mIU/mL
6 hours		**4.5 nmol/L (∼130 ng/dL)**	8.7 IU/L; mIU/mL
8 hours		3.2 nmol/L (∼92 ng/dL)	5.6 IU/L; mIU/mL
24 hours		1.5 nmol/L (∼43 ng/dL)	5.4 IU/L; mIU/mL
48 hours		0.8 nmol/L (∼23 ng/dL)	1 IU/L; mIU/mL
72 hours			1 IU/L; mIU/mL
**Case 2**	Baseline	**6.6 nmol/L (∼190 ng/dL)**	6.4 IU/L; mIU/mL
2 hours		**6.2 nmol/L (∼179 ng/dL)**	4 IU/L; mIU/mL
4 hours		**4.8 nmol/L (∼138 ng/dL)**	2.3 IU/L; mIU/mL
6 hours		**2.8 nmol/L (∼81 ng/dL)**	2.6 IU/L; mIU/mL
24 hours		1 nmol/L (∼29 ng/dL)	1.2 IU/L; mIU/mL
48 hours		0.8 nmol/L (∼23 ng/dL)	0.8 IU/L; mIU/mL
72 hours			<0.5 IU/L; mIU/mL

Abnormal values are shown in bold font. Values in parentheses are reference range in International System of Units (SI).

References range quoted for post-menopausal women. Conversion: testosterone ng/dL = nmol/L × 28.84. LH: 1 IU/L = 1 mIU/mL (numerically identical).

Abbreviations: GnRH, gonadotropin-releasing hormone; LH, luteinizing hormone.

### Case 2

The patient was initially reluctant to proceed with immediate surgery; therefore, a single dose of GnRH antagonist (degarelix) was administered. This led to rapid total testosterone reduction from 6.6 nmol/L [SI: 190 ng/dL] to 0.8 nmol/L [SI: 23 ng/dL] within forty-eight hours (reference range, <2.4 nmol/L [SI: <70 ng/dL]), confirming gonadotropin dependence (Table 1). After the initial dose, monthly GnRH antagonist injections were continued.

## Outcome and follow-up

### Case 1

The patient reported marked improvement in hirsutism, fewer headaches, fatigue, and reduced scalp hair thinning. While headaches are often incidental, such symptoms can occasionally correlate with the systemic effects of severe hyperandrogenism or related polycythemia. The patient tolerated the GnRH antagonist well with no vasomotor symptoms and expressed a desire to continue with degarelix 80 mg monthly. Total testosterone levels have consistently remained below 1.0 nmol/L (SI: <29 ng/dL) since initiating this drug. She continued monthly degarelix injections for six months, after which the regimen was switched to three-monthly dosing, which she has maintained to date with no adverse features. We continue to review three-monthly as an outpatient. No follow-up imaging was arranged, as the patient declined both surgical intervention and further radiological assessment.

### Case 2

**Figure 3:**
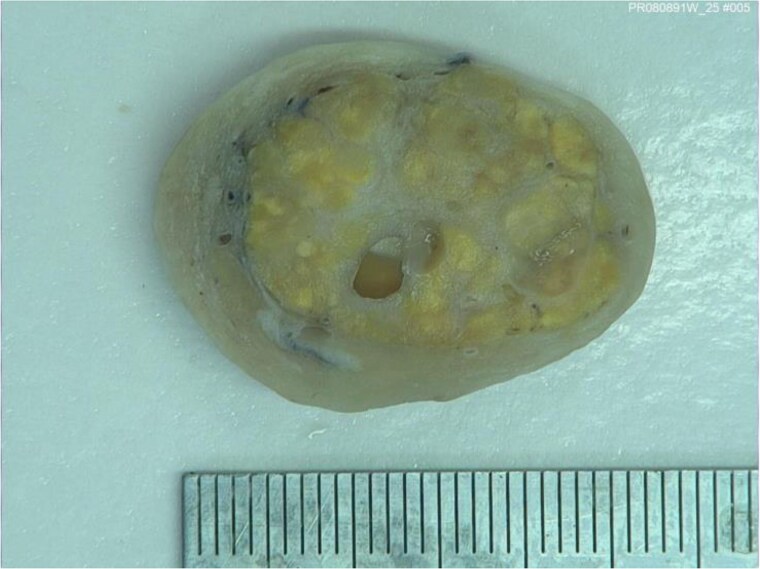
Macroscopic assessment revealed an ill-defined yellow/orange solid and cystic lesion.

**Figure 4:**
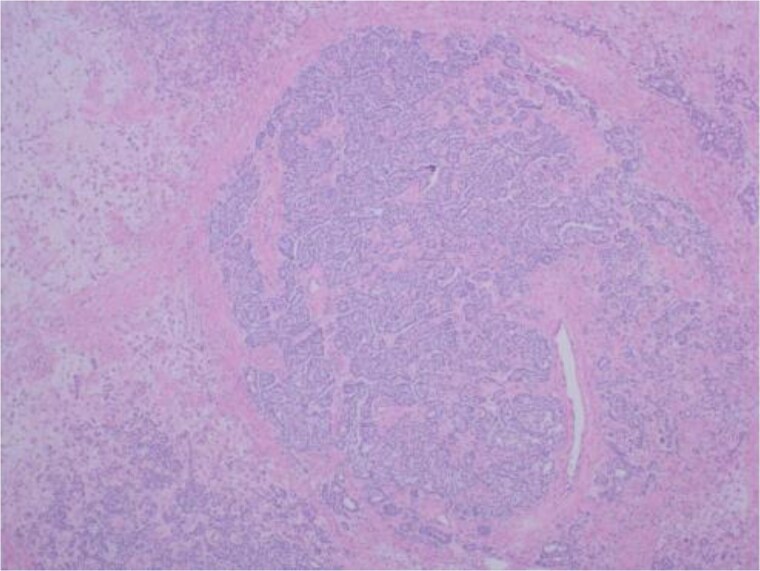
Tumor on low power. ×40 magnification. Basaloid cells arranged in solid, nested, and glandular arrangements represent the Sertoli cell component. Polygonal cells with eosinophilic cytoplasm represent the Leydig cell component.

**Figure 5:**
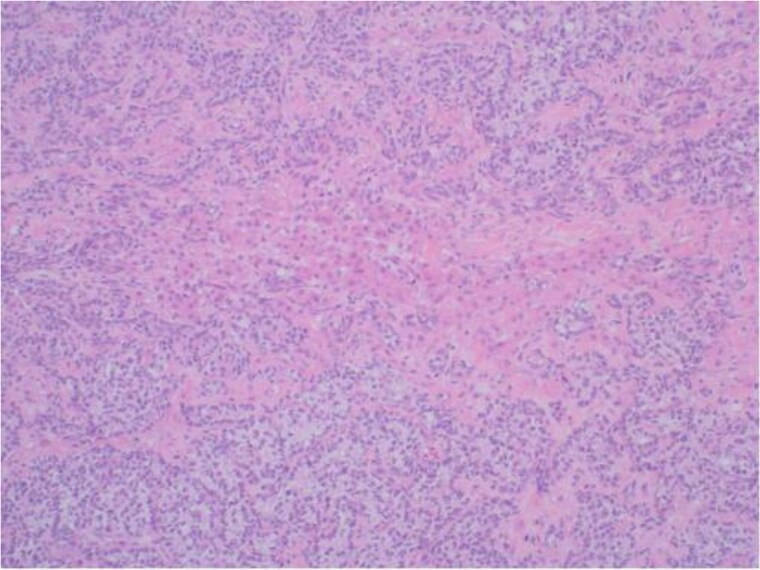
High-power images of the tumor. ×200 magnification. The basaloid cells representing the Sertoli cell component are relatively uniform with scant cytoplasm and focally angulated nuclei. The Leydig cell component contains groups of round, polygonal cells with prominent nucleoli and abundant eosinophilic cytoplasm.

**Figure 6:**
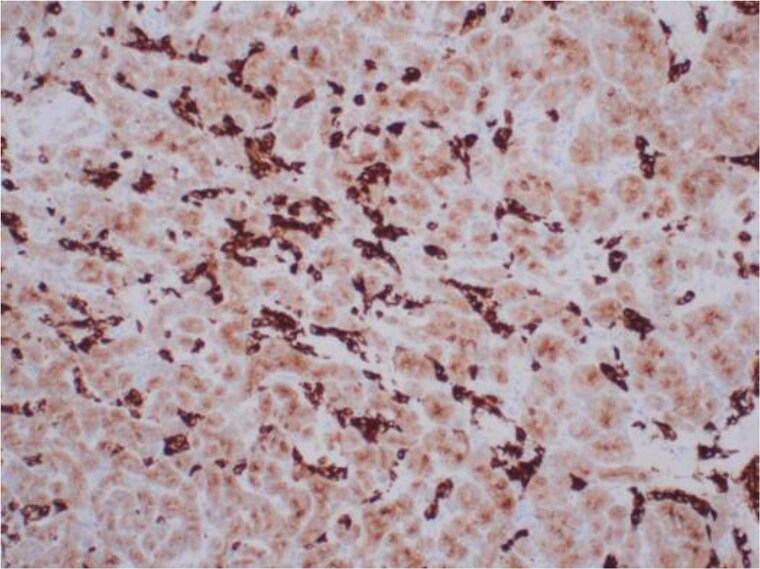
×400 magnification. Immunohistochemistry showed expression of the peptide hormone inhibin within both components, but with weaker expression in the Sertoli cell components. Inhibin has a role in distinguishing sex cord stromal tumors from other types of ovarian neoplasms [8].

Although there was no initial improvement in mood swings and hirsutism, after five months of GnRH antagonist, symptoms improved. There were mild side effects, including chills and sweats, likely relating to the GnRH antagonist injections. The patient opted for definitive surgical removal of the ovarian mass and underwent total abdominal hysterectomy and bilateral salpingo-oophrectomy (TAH & BSO), and 3 months post-operatively, and thereafter, symptom resolution has been permanent. Histological assessment of the left ovary revealed a 30 mm yellow/orange ill-defined solid lesion abutting the capsule containing a small unilocular cyst (Fig. 3). Tissue histopathology with hematoxylin and eosin staining showed two different cellular components, suggestive of a Sertoli-Leydig cell tumor (Figs. 4 and 5). The tumor cells expressed AE1/3 and Inhibin (Fig. 6) on immunohistochemistry, confirming the diagnosis of a Sertoli-Leydig cell tumor.

## Discussion

We describe two cases of GnRH antagonist-responsive ASOTs, in which treatment with a GnRH antagonist supported both diagnosis and management. ASOTs are rare but important causes of severe hyperandrogenism. Although surgical excision remains the definitive treatment, GnRH antagonists may provide a useful diagnostic adjunct and temporary therapeutic option. A previously reported 58-year-old woman with clinical and biochemical hyperandrogenism and no adrenal or ovarian lesion on imaging was similarly treated with a depot GnRH antagonist, achieving sustained testosterone suppression and clinical improvement without surgery, consistent with our findings [9]. Beyond ASOTs, evidence for GnRH antagonist use in ovarian malignancy without surgical intervention is extremely limited. We identified one Phase II study evaluating the use of the GnRH antagonist cetrorelix in patients with confirmed ovarian malignancy who did not undergo surgery. Treatment was associated with suppression of luteinizing hormone; however, androgen levels, including testosterone, were not measured or reported. Consequently, the effect of GnRH antagonism on testosterone in this clinical context remains unclear [10].

While surgery remains standard for ASOTs, medical therapy with GnRH analogs has been used in select cases. GnRH antagonists directly block pituitary GnRH receptors, causing immediate suppression of LH and FSH and rapid reduction in ovarian steroidogenesis. This differs from GnRH agonists, which may initially trigger a gonadotropin flare. The absence of flare makes antagonists particularly attractive in severe virilization, where rapid biochemical response may support localization of androgen excess [11].

The proposed rationale for GnRH analog suppression testing is based on gonadotropin dependence of androgen secretion. Benign disorders such as ovarian stromal hyperthecosis are typically LH-dependent and often show marked testosterone suppression. In contrast, ASOTs have traditionally been regarded as autonomous and LH-independent, particularly when malignant. However, real-world evidence suggests significant overlap. Several ASOTs, including Sertoli–Leydig cell tumors and steroid cell tumors, may retain functional gonadotropin receptors and demonstrate partial or marked testosterone suppression, while benign pathology may occasionally fail to suppress. This limits suppression testing as a discriminator between benign and malignant disease.

Ovarian stromal hyperthecosis is a benign non-neoplastic condition characterized by luteinized stromal cells producing excess androgens [12]. Sertoli-Leydig cell tumors are typically unilateral neoplasms, with testosterone secretion driven by the Leydig cell component [8, 13]. These tumors may be benign or malignant, with malignant behavior more likely in poorly differentiated tumors or those showing necrosis, increased mitotic activity, capsule rupture, or heterologous elements [13]. In our case, histology was consistent with a well-differentiated (Grade 1) tumor without heterologous elements or necrosis, supporting benign behavior.

In our cases, total testosterone fell by 89% (Case 1) and 85% (Case 2) within twenty-four hours of GnRH antagonist administration. Based on these findings and previous reports demonstrating rapid and sustained testosterone suppression [9], we propose that a > 50% reduction in total testosterone within twenty-four hours may suggest benign or well-differentiated ovarian pathology. A possible mechanism is that well-differentiated tumors retain LH receptor expression and remain partially hypothalamic pituitary gonadal (HPG) axis dependent, whereas malignant tumors may become more autonomous.

However, this proposed threshold must be interpreted cautiously. Current evidence is insufficient to validate a 50% reduction in total testosterone as a reliable discriminator. Response may also be influenced by dosing, timing of sampling, baseline gonadotropin status, and menopausal state. Additionally, GnRH receptors may be expressed in some gynecological tumors, raising the possibility of direct ovarian effects independent of pituitary suppression, further complicating interpretation. GnRH antagonist testing should therefore be viewed as an adjunctive tool. Testosterone suppression may support an ovarian source but cannot reliably distinguish hyperthecosis from virilizing ovarian tumors, nor can it exclude malignancy.

Delaying surgery carries risk, particularly in postmenopausal women with rapidly progressive virilization, where lack of suppression raises concern for occult malignancy and warrants prompt surgical exploration. Conversely, even when suppression is observed, prolonged reliance on medical therapy may delay definitive treatment of malignant disease.

While surgery remains the gold standard, GnRH antagonists may have a role as a short-term diagnostic adjunct, bridging therapy prior to surgery, or longer-term treatment in patients unfit for surgery or who decline intervention, provided close biochemical and clinical monitoring is performed.

Further studies are required to establish whether GnRH antagonist suppression testing can reliably localize the androgen source and stratify malignancy risk. These cases highlight the potential role of GnRH antagonists in severe female hyperandrogenism. Although >50% testosterone suppression within twenty-four hours may suggest benign or well-differentiated disease, surgery remains essential for definitive diagnosis and management of suspected ASOTs.

In conclusion, these cases demonstrate the potential role of GnRH antagonists in the diagnosis and treatment of ovarian-driven hyperandrogenism, particularly when surgery is not immediately feasible or preferred.

## Learning points

- GnRH antagonists may be helpful in the evaluation and management of ovarian hyperandrogenism.
- GnRH antagonist diagnostic administration may serve as a non-invasive tool to determine gonadotropin dependency of hyperandrogenism and stratify risk.
- Further studies are needed to assess long-term outcomes in treatment with GnRH antagonist in the setting of female hyperandrogenism.

## Data Availability

Data sharing is not applicable to this article as no datasets were generated or analysed during the current study.
